# A Rare Presentation of a Periorbital Herpes Simplex Virus Pseudotumor in an Immunocompromised Patient

**DOI:** 10.7759/cureus.64860

**Published:** 2024-07-18

**Authors:** John M Sousou, Vincent Santi, Abdullah Mohamed, Reeba Omman, Jorge Verdecia

**Affiliations:** 1 Internal Medicine, University of Florida College of Medicine – Jacksonville, Jacksonville, USA; 2 Infectious Disease, University of Florida College of Medicine – Jacksonville, Jacksonville, USA; 3 Pathology and Laboratory Medicine, University of Florida College of Medicine – Jacksonville, Jacksonville, USA

**Keywords:** ocular herpes, immunocompromised patient, human immunodeficiency virus (hiv) infection, herpes simplex infection, hsv pseudotumor

## Abstract

Herpes simplex virus (HSV) frequently affects the ocular and genital regions, especially in immunocompromised individuals. On rare occasions, HSV infections can present as pseudotumors. These pseudotumors may mimic cancerous growths, condylomas, or hypertrophic lesions rather than the characteristic small ulcerations. The development of pseudotumors due to HSV is particularly uncommon, especially in the facial region. This atypical presentation poses significant diagnostic challenges and may potentially lead to erroneous identification as a cancerous growth. This case report details a 53-year-old African American man with human immunodeficiency virus (HIV) (noncompliant with antiretroviral therapy) presenting with a purulent ocular pseudotumor secondary to HSV infection, along with a review of the literature surrounding HSV pseudotumors.

## Introduction

Herpes simplex virus (HSV) can result in painful ocular sores, potentially leading to vision impairment if left untreated. Herpes simplex virus type 1 (HSV-1), the predominant subtype that causes ocular herpes, typically manifests as painful and recurrent sores on the eyelids, conjunctiva, or cornea [1]. The virus gains access to ocular tissues through direct contact with infected secretions or compromised skin, leading to primary infection. However, most ocular diseases caused by HSV result from the reactivation of a dormant state when the virus infiltrates sensory neurons of the trigeminal nerve ganglia [2]. Herpetic lesions in the eye can range from mild epithelial keratitis or blepharitis to more severe forms, such as stromal keratitis and necrotizing retinitis, potentially resulting in vision impairment or blindness if left untreated.

In rare cases, patients with uncontrolled human immunodeficiency virus (HIV) can develop atypical masses or nodular lesions, referred to as pseudotumors, secondary to HSV infection [3]. HSV primarily targets the oral and anogenital lesions; the most common location where these pseudotumors may arise is the anogenital region. However, a few reported cases of facial lesions exist in the literature [4]. This case details an uncommon presentation of a facial HSV pseudotumor in an immunocompromised individual. 

## Case presentation

**Figure 1:**
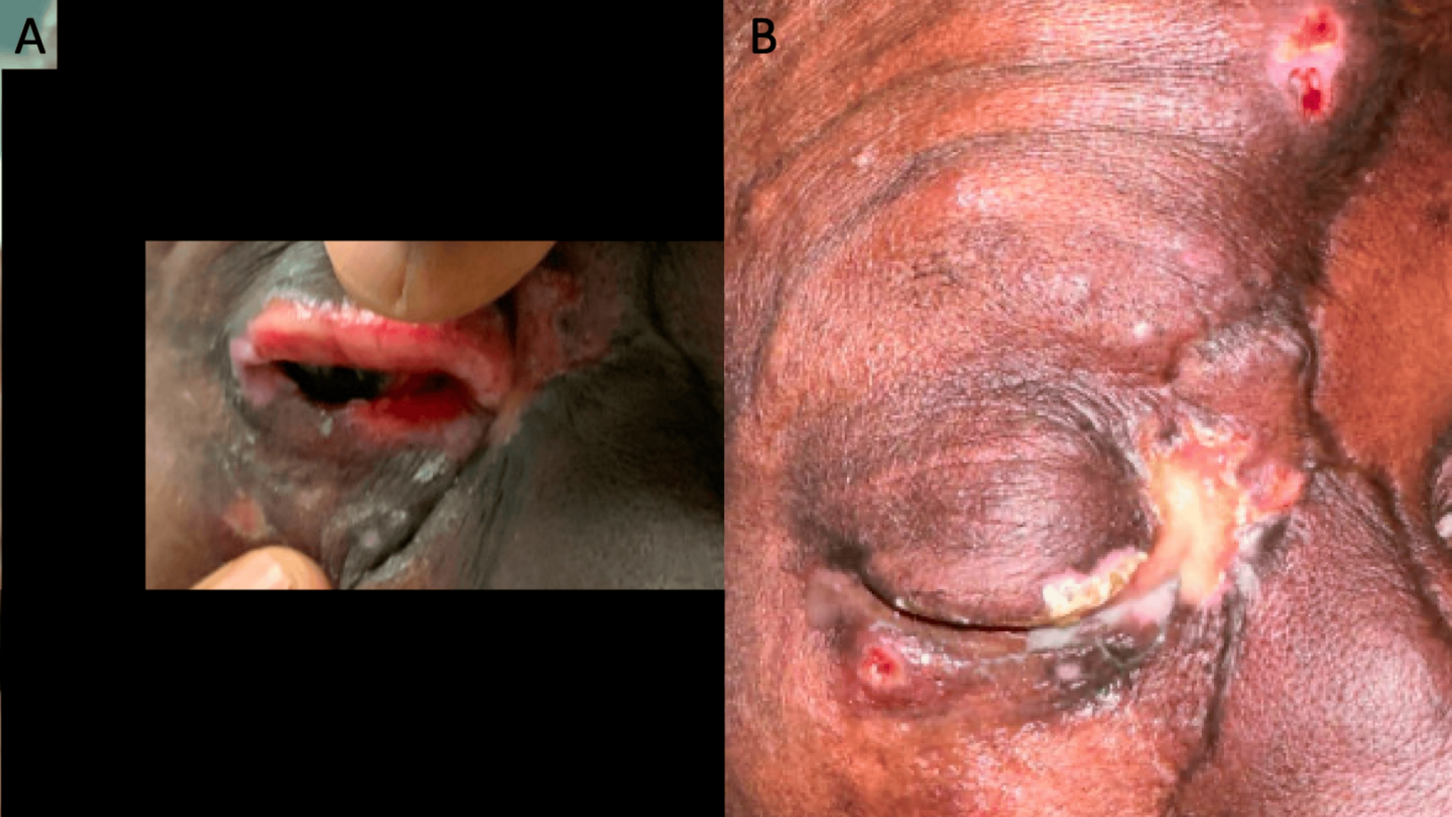
Photographs of the patient's eye one month prior to admission (A) and on the day of admission (B).

**Figure 2:**
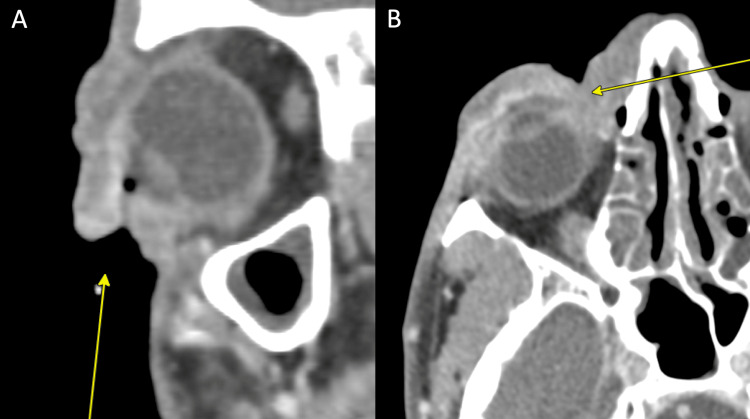
Coronal (A) and axial (B) CT images of the maxillofacial regions demonstrating soft tissue swelling over the left frontal bone and left temporal region. CT: computed tomography

A 53-year-old African American man presented to the ED with a lesion around his right eye that had worsened over the last three weeks before admission. He has a past medical history of uncontrolled HIV with a CD4 count of 17 and multiple incarcerations over the previous 10 years. The patient reports worsening right eye swelling, redness, and occasional discharge. His initial symptoms around one month prior included periorbital erythema, edema, and discoloration (Figure 1A). Physical examination revealed distortion of his eyelid architecture with an edematous right eye and a purulent lesion around his inner eyelid with underlying nodular growth, a small ulcer superiorly on his forehead, and a small ulcer on his inferior eyelid (Figure 1B). Initial laboratory testing was notable only for a 16.5 × 109/L leukocytosis. Ophthalmology was consulted, who saw the patient and noted 20/100 vision in the right eye compared to 20/40 in the left eye. They noted depigmentation and ulceration of the upper and lower eyelids with destruction of the eyelashes; no corneal findings were noted, and no evidence of keratitis, exposure keratopathy, or lagophthalmos was noted. Ophthalmology ultimately noted an unclear etiology, but recommended the administration of neomycin/polymyxin/dexamethasone ointment three times a day to the right eye for seven total days. His maxillofacial region's computed tomography (CT) scan revealed diffuse soft tissue thickening along the right periorbital region extending to the right premaxillary tissues and right nasal tissues with adjacent conjunctival enhancement (Figure 2).

**Figure 3:**
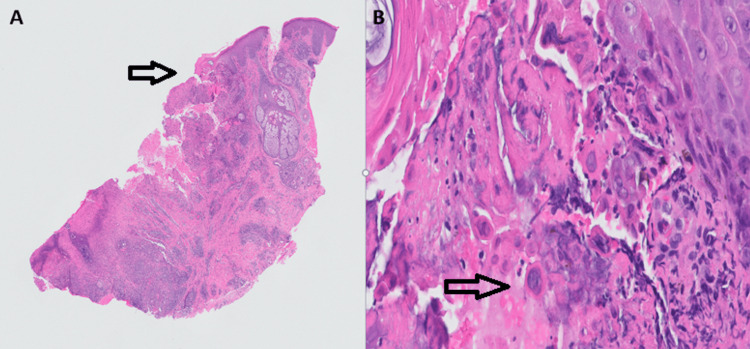
Skin biopsy of the right upper eyelid with nose junction (×2) showing cutaneous ulceration with marked inflammation (arrow) (A). High-power picture (×40) showing viral inclusions and the cytopathic effect of multinucleation, nuclear molding, and margination of the chromatin (arrow) (B).

Infectious workup, including blood cultures, respiratory cultures, respiratory viral panels, fungal cultures, QuantiFERON gold, syphilis, and *Cryptococcus*, were all negative. A swab of the patient's right upper eyelid/nose junction lesion was sent for polymerase chain reaction (PCR) testing and was positive for HSV-1. A biopsy of the same lesion was then taken, which revealed cutaneous ulceration with inflammation and viral inclusions, confirming the presence of herpes virus (Figure 3).

The patient was subsequently initiated on valacyclovir 1,000 milligrams every 12 hours for 14 days. He was scheduled to follow up in the infectious disease clinic within two weeks after discharge; however, he was ultimately lost to follow-up and was unable to be contacted as he is currently homeless.

## Discussion

This case highlights a rare presentation of a facial HSV pseudotumor in an immunocompromised patient with untreated HIV. HSV typically affects ocular and perioral regions, leading to various degrees of ocular disease ranging from mild keratitis to severe necrotizing retinitis. The development of pseudotumors due to HSV, particularly in the facial region, is an unusual manifestation, generally associated with significant immunosuppression. Their atypical presentation may lead to diagnostic challenges due to their appearance. Pseudotumors typically appear as nodules resembling tumors, condylomas, or hypertrophic lesions rather than a typical ulcer, potentially leading to erroneous identification as cancerous growths [5].

Diagnostic challenges were evident in this case. The patient's initial presentation with periorbital erythema, edema, and subsequent development of ulcerative lesions prompted a comprehensive infectious workup. The negative results for a broad range of infections, including bacterial, fungal, and other viral pathogens, highlighted the importance of considering HSV in the differential diagnosis. The definitive diagnosis was achieved through PCR testing and histopathological examination. 

The primary treatment of HSV-associated ocular or facial disease includes oral agents such as acyclovir or valacyclovir to inhibit viral replication and reduce the severity and duration of ocular symptoms. Topical agents, including acyclovir ointment, ganciclovir gel, and trifluridine drops, may also be used for milder cases [6]. Topical glucocorticoids as monotherapy should be avoided due to the risks of exacerbating viral replication or the possibility of involving deeper stromal structures, which could lead to vision loss [7]. In severe or recurrent cases of HSV ocular disease, systemic antiviral therapy may be considered to provide broader viral suppression and prevent complications. In rare cases of severe HSV keratitis with corneal scarring or perforation, surgical procedures such as corneal transplantation may be necessary to restore vision and ocular integrity.

## Conclusions

This case highlights the importance of considering HSV infection in patients presenting with ocular manifestations, particularly in immunocompromised individuals such as our patient with a history of uncontrolled HIV. Pseudotumors caused by HSV may resemble malignant tumors in their clinical presentation and imaging findings, which can potentially lead to misdiagnosis and inappropriate treatment if not properly identified. Correctly identifying HSV pseudotumors allows for the appropriate administration of antiviral therapy rather than surgical or oncological treatments, resulting in better patient outcomes.
